# Distributed activity in the human posterior putamen distinguishes goal-directed from habitual control in humans

**DOI:** 10.1101/2025.11.26.690863

**Published:** 2025-11-28

**Authors:** Amogh Johri, Lisa Kluen, Rani Gera, Vincent Man, Omar Perez, Julia Pia Simon, Weilun Ding, Aniek Fransen, Scarlet Cho, Sarah Oh, Jeff Cockburn, Jamie D. Feusner, Reza Tadayonnejad, John P. O’Doherty

## Abstract

There is considerable variability across individuals in the behavioral expression of goal-directed and habitual control, and yet little is known about the underlying functional neuroanatomical basis of such differences. Here, we aimed to determine whether distinct patterns of brain activity during initial learning of instrumental actions can indicate whether an individual subsequently utilizes a goal-directed or habitual behavioral strategy. We applied multivariate pattern analyses to a large fMRI dataset composed of 108 participants performing an instrumental action-learning task. Region-of-interest (ROI) and whole-brain searchlight analyses with cross-validation indicate that distributed activation patterns in the left posterior putamen during training distinguish goal-directed from habitual participants, ascertained via sub-sequent administration of an outcome devaluation test. This finding was further replicated and generalized to two fully held-out samples: an additional cohort of healthy participants (n = 38) and a separate group of psychiatric patients with variable diagnoses (n = 55). These findings reveal that it is possible to predict an indi-vidual’s overall behavioral strategy from circumscribed activity in the human brain, while specifically implicating the posterior putamen in the implementation of distinct behavioral control strategies.

